# HIN5/439: A Web-based Information Sharing System for Patient Referral

**DOI:** 10.2196/jmir.1.suppl1.e36

**Published:** 1999-09-19

**Authors:** L Jae Ok, M Won-Ki, K Mi Na, K Won-Sub, L Kyoung Soo, L Tae-Hwan, C Han Ik

**Affiliations:** ^1^Ajou UniversitySuwonSouth Korea

**Keywords:** Health Care Delivery, World wide web, Information system

## Abstract

**Introduction:**

It is essential to have a proper health care delivery system for the efficient management of clinical resources for a nation. In Korea, we have observed numerous problems of missing or delaying clinical information when referring patients to a nearby secondary or tertiary care hospital. Patient referral is usually done at random but no reliable information is provided for appropriate care. Furthermore, no follow-up information is sent back to referring institutions when the care is completed such as discharge summary. The aim is therefore to develop and implement a Web-based information sharing system to facilitate health care delivery during the course of referring patient transfer.

Materials and Method:

Researchers first conducted a survey with clinicians to extract clinical expertise and relevant information to standardize a core set of information service protocols for sharing through the internet. Then, a model was proposed to distribute and share intra-hospital database among consenting networked clinicians around the region. The core set of protocols includes textual reports of laboratory test results, and imaging reports of endoscopy and diagnostic radiology procedures. Also, therapeutic and medication reports are also included.

**Results:**

The Web-based information sharing service is currently in use at patient referral center in Asian Medical Center, a tertiary care teaching hospital located in Seoul, Korea. So far, 11,587 patients were serviced in 1998. During hospitalization, each referring physician is given access code to check status of the referred patient. When the patient is discharged or transferred back to the referring institute, discharge summary and other adequate information are provided through simple file transfer.

**Discussion:**

We are eager to report the first implementation of a Web-based information sharing system for efficient patient referral services in Korea. Though an evaluation of the system performance is still in progress, we have found the system practically usable and cost-effective in clinical environment. Therefore we suggest that a web based information sharing technique would be a method of choice for improving the management and efficiency of health care delivery system.

